# *Candida blankii*: an emergent opportunistic yeast with reduced susceptibility to antifungals

**DOI:** 10.1038/s41426-017-0015-8

**Published:** 2018-03-07

**Authors:** João Nobrega de Almeida, Silvia V. Campos, Danilo Y. Thomaz, Luciana Thomaz, Renato K. G. de Almeida, Gilda M. B. Del Negro, Viviane F. Gimenes, Rafaella C. Grenfell, Adriana L. Motta, Flávia Rossi, Gil Benard

**Affiliations:** 1 Central Laboratory Division– LIM-03, Hospital das Clínicas da Faculdade de Medicina da Universidade de São Paulo, São Paulo, 05403-010 Brazil; 2 Laboratory of Medical Mycology - LIM-53, Hospital das Clínicas FMUSP and Instituto de Medicina Tropical de São Paulo, Universidade de São Paulo, São Paulo, 05403-000 Brazil; 3 Heart Institute, Hospital das Clínicas da Faculdade de Medicina da Universidade de São Paulo, São Paulo, 05403-000 Brazil; 4 Institute of Biomedical Science, Universidade de São Paulo, São Paulo, 05508-900 Brazil; 5 Department of Biophysics, Escola Paulista de Medicina, Universidade Federal de São Paulo, São Paulo, 04039-000 Brazil

In 1968, Buckley and van Uden described the non-fermenting yeast *Candida blankii* (*C. blankii*) found in the organs of a mink^1^. Until recently, this microorganism had only been the subject of biotechnological research^2,3^. However, in 2015, Zaragoza et al. reported a 14-year-old male patient with cystic fibrosis (CF), who had pulmonary exacerbations with repeated isolation of *C. blankii* from respiratory samples^4^. This finding raised the hypothesis that this yeast could be a relevant pathogen for CF patients^4^.This paper corroborates this initial observation by describing a bloodstream infection by *C. blankii* in a CF patient who underwent lung transplantation.

A 16-year-old female with CF was referred to our lung transplant center in March 2016. Her recent medical history was notable for severe pulmonary exacerbations, which required prolonged hospitalization and mechanical ventilation. Her most recent preadmission sputum cultures collected by the referring hospital showed positive for *Staphylococcus aureus, Pseudomonas aeruginosa, Burkholderia cepacia, Aspergillus sp*., including positive samples for *Candida* sp. (negative germ tube, isolates not available) collected after itraconazole therapy (200 mg/day) used to treat the pulmonary exacerbations. In June 2016, she underwent bilateral lung transplantation at the Hospital das Clínicas, University of São Paulo, Brazil, under antimicrobial prophylaxis consisting of teicoplanin, meropenem, cotrimoxazole and liposomal amphotericin B (L-AMB, 200 mg/day). However, during the infusion of L-AMB, the patient presented hypotension, leading to the discontinuation of the antifungal treatment. On the first postoperative day (POD), the patient developed sepsis, and so blood cultures were collected (Bactec aerobic and anaerobic/Plus, BD). Peripheral and central venous catheter blood cultures became positive for yeasts after 41 (anaerobic bottle) and 72 (aerobic bottles) hours of incubation, respectively. On the third POD, due to the provisional report of yeasts on blood cultures, micafungin (100 mg/day) was prescribed. Blood cultures collected 72 h after the introduction of micafungin became negative; transthoracic echocardiography and fundoscopic eye exam did not show any significant results. The yeast isolate showing pale pink colonies on chromogenic medium (BBL CHROMagar, BD, Sparks, USA) were not identified by MALDI-TOF mass spectrometry (Vitek MS™, IVD library, bioMérieux, Marcy-L´Etoile, France). Because clinical improvement was observed, micafungin was maintained for 14 days. The patient was discharged on the 39th POD.

The clinical isolate (HCFMUSP01) was later identified as *C. blankii* after sequence analysis of the internal transcribed spacer 1 (ITS1, GenBank accession no. MF573785) and D1D2 region from the 26S subunit (D1D2, GenBank accession no. MF940140) of the rRNA^5,6^. Since little is known about this microorganism as an opportunistic pathogen, we further characterized this species in terms of genetic and proteomic diversity, antifungal susceptibility, biofilm production, and in vivo virulence by analyzing the clinical isolate and the strains (IIC1M.1, BX90C, BX81A) from the yeast collection at the Federal University of Minas Gerais, Brazil.

The organisms had identical D1D2 rRNA sequences, but the clinical isolate showed a different ITS allele (single nucleotide deletion) when compared to the other strains (Table 1 and supplementary Table S1). Proteomic analysis by MALDI-TOF mass spectrometry (MS), this time using both Bruker’s (Microflex™, Bruker Daltonics, Bremen, Germany) and bioMérieux instruments (Vitek MS™, bioMérieux, Marcy L’Etoile, France), revealed distinct spectral profiles between clinical and environmental organisms (Supplementary Figure S1). As an observation, the Bruker’s IVD and RUO libraries lacked this species and were unable to identify the organisms, whilst the Vitek MS™ RUO library correctly identified all of them (Table 1).

**Table 1:** Allelic diversity, proteomic species identification, and antifungal susceptibility testing of *Candida blankii* organisms

Strain name	GenBank accession no. (ITS; D1/D2)	ITS allele	Correct species identification by MALDI-TOF mass spectrometry				Antifungal susceptibility testing (minimal inhibitory concentration)				
			Bruker IVD^a^ Library	Bruker RUO^b^ Library	Vitek MS IVD Library	Vitek MS RUO Library	Flu^c^	Vor^d^	AmB^e^	Anf^f^	Mif^g^
HCFMUSP01	MF573785.1; MF940140	A	No	No	No	Yes	16	0.5	0.5	1	0.5
IIC1M.1^h^	MF940143; MF940137	B	No	No	No	Yes	16	0.5	0.25	0.25	0.25
BX 90C^i^	MF940141; MF940139	B	No	No	No	Yes	16	0.5	0.5	0.25	0.25
BX 81A^i^	MF940142; MF940138	B	No	No	No	Yes	16	0.5	0.5	0.25	0.25

^a^ In vitro diagnostics. ^b^ Research use only. ^c^ Fluconazole. ^d^ Voriconazole. ^e^ Amphotericin B. ^f^ Anidulafungin. ^g^ Micafungin. ^h^ Sands of Copacabana’s beach, Rio de Janeiro, Brazil. ^i^ Sugarcane bagasse, Paraíba, Brazil

Antifungal susceptibility testing was carried out using the EUCAST reference broth microdilution method^7^. All organisms showed high minimal inhibitory concentrations (MICs) of fluconazole (Sigma-Aldrich, St. Louis, MO, USA) (16 mg/L) and voriconazole (Sigma-Aldrich) (0.5 mg/L), whereas amphotericin B (Sigma) exhibited potent activity (0.25–0.5 mg/L). Both anidulafungin (Pfizer, Groton, CT, USA) and micafungin (Astellas Pharma US, Northbrook, IL, USA) showed limited activity against *C. blankii* organisms, with MICs raging from 0.25–1 mg/L and 0.25–0.5 mg/L, respectively (Table 1).

Biofilm formation and metabolic activity were measured by using crystal violet staining^8^ and XTT reduction assay^9^, respectively. Based on previously reported cutoff values^10^, all organisms were classified as low biofilm producers, showing low metabolic activity (Supplementary Figure S2).

Killing assays in *Galleria mellonella* model were performed to compare the pathogenicity of *C. blankii* organisms with *Candida albicans* (strain SC5314)^11^. Sixteen *G. mellonella* larvae (LFDP/ICB/USP) were used for each test group. Larvae were inoculated (1 × 10^5^ and 1 × 10^6^ cells/larva) and incubated at 37 °C. Pathogenicity was assessed, monitoring the percent survival over 7 days and plotted using Kaplan–Meier and the Log-rank test (GraphPadPrism 4, GraphPad Software, Inc., La Jolla, USA). The *C. blankii* organisms HCFMUSP01 and IIC1M.1 were significantly less virulent than the *C. albicans* strain (*P* < 0.0001) in the *G. mellonella* model (Supplementary Figure S3). However, the strains BX 90C and BX 81 A showed similar pathogenicity when compared to *C. albicans* (*P* = 0.13). Moreover, *G. mellonella* killing by different *C. blankii* organisms were accentuated when larvae were inoculated with 1 × 10^6^ organisms (Supplementary Figure S3).

Evidence is provided here that *C. blankii* is an opportunist pathogen for lung transplant and/or CF patients. Clinical laboratories from referral centers of CF and lung transplant should be aware of this *Candida* species, and the fact that not all MALDI-TOF MS libraries are able to identify it. Based on our case report and the in vitro data presented here, we suggest that CVC was not the primary source of the infection and that the patient’s respiratory tract was heavily colonized by *C. blankii* (assuming that the *Candida* sp. from the referring hospital was *C. blankii* and based on *G. mellonella* killing assays). This probably led the patient to develop fungemia due to the lack of antifungal prophylaxis.

Further studies of different strains are warranted to increase knowledge of genetic diversity and antifungal susceptibility profile of *C. blankii* organisms. However, while more microbiological data are pending, it is prudent to avoid azoles for the treatment or prophylaxis of *C. blankii* infections. Like *Candida parapsilosis*, despite the reduced antifungal action of echinocandins against *C. blankii*, successful treatment may be achieved with these compounds, as illustrated in this report. However, amphotericin B showed strong in vitro activity against *C. blankii*, and its formulations should be the first line therapy for deep-seated infections by this emergent species while antifungal susceptibility testing is ongoing.

## Electronic supplementary material

Supplementary Table S1

Supplementary Figure S1

Supplementary Figure S2

Supplementary Figure S3
