# Supplementary material for: Candida blankii: an emergent opportunistic yeast with reduced susceptibility to antifungals
Source: Emerg Microbes Infect. 2018 Mar 7;7:24. doi: 10.1038/s41426-017-0015-8 (PMC5841406; doi:10.1038/s41426-017-0015-8)
Supplement: Supplementary file 1 — Supplementary Table S1 [file 41426_2017_15_MOESM1_ESM.docx]

**Supplementary material Table S1**. Representative ribosomal internal transcribed spacer rDNA sequences from *Candida blankii*, GenBank access numbers, including country and source of isolation, and haplotypes diversity

| **Strain** | **NCBI access No.** | **Country** | **Source** | **Single nucleotide**  **polymorphism (5’-3’)** |
| --- | --- | --- | --- | --- |
| BX 90C | MF940141 | Brazil | Environmental, sugarcane bagasse | AAATTTGACCTCA |
| BX 81A | MF940142 | Brazil | Environmental, sugarcane bagasse | AAATTTGACCTCA |
| IIC1M.1 | MF940143 | Brazil | Environmental, soil | AAATTTGACCTCA |
| HCFMUSP01 | MF573785 | Brazil | Clinical, human blood | AAATT- GACCTCA |
| CBS7205 | KY101964.1 | New Zealand | Animal, uterus of horse | AAATTTGACCTCA |
| CBS6734 | KY101963.1 | Japan | Environmental, soil | AAATT- GACCTCA |
| CBS6427 | [KY101968.1](https://www.ncbi.nlm.nih.gov/nucleotide/1102638398?report=genbank&log$=nuclalign&blast_rank=1&RID=VV7X7E4K014) | Japan | Environmental, soil | AAATT- GACCTCA |
| CBS1898 | KY101966.1 | Canada | Animal, gut of mink | AAATTTGACCTCA |
| PMM08 753L | KP131678.1 | France | Clinical | AAATTTGACCTCA |
