# Supplementary material for: Candida blankii: an emergent opportunistic yeast with reduced susceptibility to antifungals
Source: Emerg Microbes Infect. 2018 Mar 7;7:24. doi: 10.1038/s41426-017-0015-8 (PMC5841406; doi:10.1038/s41426-017-0015-8)
Supplement: Supplementary file 2 — Supplementary Figure S1 [file 41426_2017_15_MOESM2_ESM.docx]

**
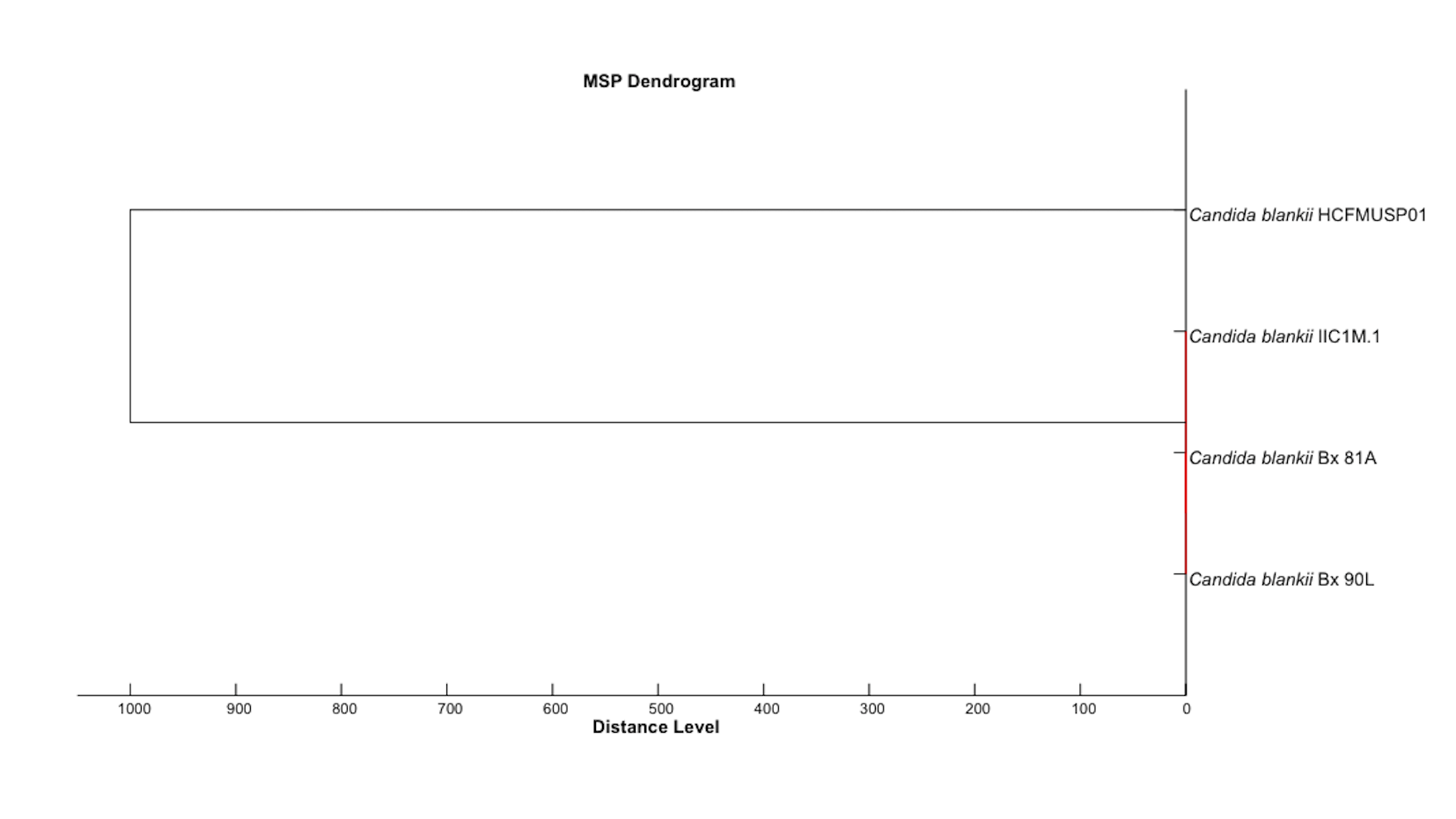
**

**Supplementary Figure S1:** Bruker Biotyper™ dendrogram clustering of *Candida blankii* organisms main spectrum profiles (MSPs). Distances were displayed in relative units on the *x* axis
