# Supplementary material for: Candida blankii: an emergent opportunistic yeast with reduced susceptibility to antifungals
Source: Emerg Microbes Infect. 2018 Mar 7;7:24. doi: 10.1038/s41426-017-0015-8 (PMC5841406; doi:10.1038/s41426-017-0015-8)
Supplement: Supplementary file 3 — Supplementary Figure S2 [file 41426_2017_15_MOESM3_ESM.docx]

**Supplementary Figure S2.** Metabolic activity of *Candida albicans* and *Candida blankii* biofilms. Bars represent metabolic activity as measured by the XTT assay and by spectrophotometry at 490 nm. Assays were performed in quadruplicate and results are presented as the mean of four replicates. Error bars represent the standard deviation of the mean
