# Supplementary material for: Candida blankii: an emergent opportunistic yeast with reduced susceptibility to antifungals
Source: Emerg Microbes Infect. 2018 Mar 7;7:24. doi: 10.1038/s41426-017-0015-8 (PMC5841406; doi:10.1038/s41426-017-0015-8)
Supplement: Supplementary file 4 — Supplementary Figure S3 [file 41426_2017_15_MOESM4_ESM.docx]

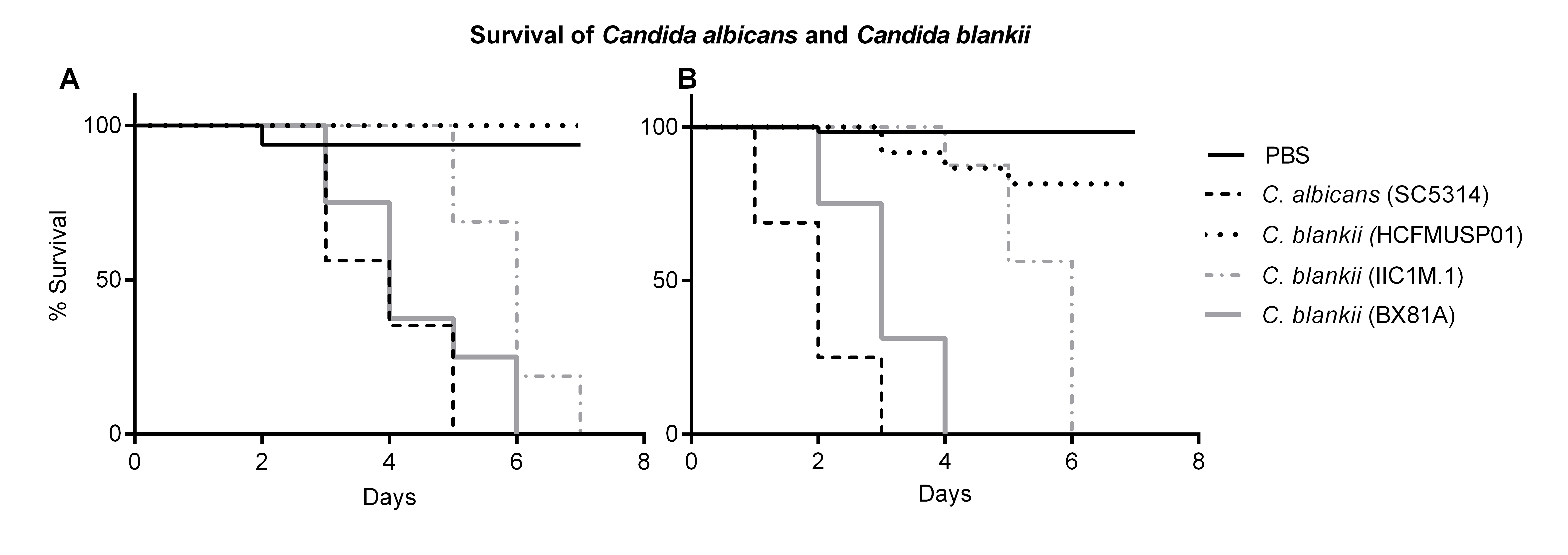


**Supplementary Figure S3.** Comparison of virulence from *Candida albicans* (control strain) and *Candida blankii,* using *Galleria mellonella* as the infection model. Graphs represent the average of three repetitions. (A) Inoculum of 1x10^5^ CFU/larvae; (B) inoculum of 1x10^6^ CFU/larvae
